# QLog Solar-Cell Mode Photodiode Logarithmic CMOS Pixel Using Charge Compression and Readout †

**DOI:** 10.3390/s18020584

**Published:** 2018-02-14

**Authors:** Yang Ni

**Affiliations:** New Imaging Technologies, Impasse de la Noisette, 91370 Verrières le Buisson CEDEX, France; yang.ni@new-imaging-technologies.com

**Keywords:** CMOS image sensor, logarithmic response, WDR, single photon detection

## Abstract

In this paper, we present a new logarithmic pixel design currently under development at New Imaging Technologies SA (NIT). This new logarithmic pixel design uses charge domain logarithmic signal compression and charge-transfer-based signal readout. This structure gives a linear response in low light conditions and logarithmic response in high light conditions. The charge transfer readout efficiently suppresses the reset (KTC) noise by using true correlated double sampling (CDS) in low light conditions. In high light conditions, thanks to charge domain logarithmic compression, it has been demonstrated that 3000 electrons should be enough to cover a 120 dB dynamic range with a mobile phone camera-like signal-to-noise ratio (SNR) over the whole dynamic range. This low electron count permits the use of ultra-small floating diffusion capacitance (sub-fF) without charge overflow. The resulting large conversion gain permits a single photon detection capability with a wide dynamic range without a complex sensor/system design. A first prototype sensor with 320 × 240 pixels has been implemented to validate this charge domain logarithmic pixel concept and modeling. The first experimental results validate the logarithmic charge compression theory and the low readout noise due to the charge-transfer-based readout.

## 1. Introduction

Silicon-based low light level image sensors are critical for many low-power, low-cost, and highly integrated vision systems. Besides resolution, the high sensitivity and wide dynamic range are two key parameters for many applications. Logarithmic response pixel is particularly interesting for such applications since it can produce a very wide dynamic range directly at the pixel level without exposure accommodation or any image processing. The instantaneous light accommodation suppresses all the auto-exposure latency which is very problematic in fast-changing environments. Compared with other high dynamic range (HDR) techniques, a logarithmic sensor can give considerable system simplification—from design to final validation—thanks to its contrast indexed image sensing and predictable behaviors under uncontrollable environments.

Traditional logarithmic pixel designs are based on an exponential law nonlinear device which converts a linear photo-current produced by a photodiode into a logarithmic voltage signal. This kind of implementation, even very simple, suffers from large fixed pattern noise (FPN), image lag, and poor low light performance [1,2,3]. The large FPN comes from both the exponential law converting device and also the dark current in the photodiode which is amplified by the logarithmic law of this converting device. This FPN is light dependent and very sensitive to temperature, so is extremely difficult to compensate. The image lag comes from the impossibility to reset the pixel and the equivalent time constant of the converting device which is reversely proportional to the light intensity. The image lag is unacceptable in dim conditions or at a high framerate.

**Figure 1:**
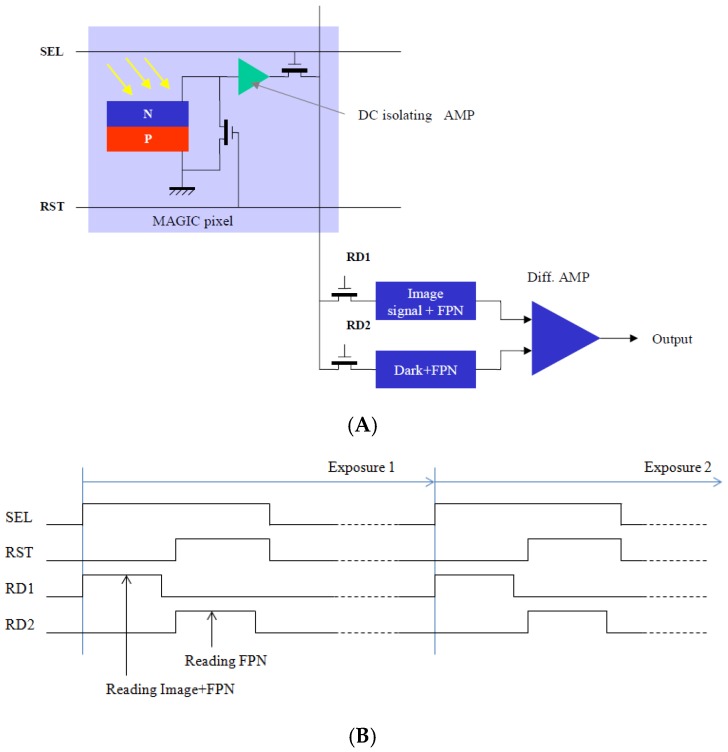
(**A**) Solar-cell mode photodiode-based logarithmic pixel and its readout chain; and (**B**) the associated control timing for readout operation with on-chip FPN compensation.

We have resolved these problems by introducing a logarithmic pixel by directly measuring the open-circuit voltage on a photodiode operating in solar-cell mode. As dictated by basic physical laws, this open-circuit voltage is naturally proportional to the logarithm of the incident light intensity on the photodiode. By the energy conservation law, we know that this open-circuit voltage should be zero when the pixel is in totally dark conditions; this gives the possibility to reset the photodiode by using a MOS transistor which simply short-circuits the photodiode. The first high-quality logarithmic CMOS sensor has been developed at Institute of Telecom in France (from where New Imaging Technologies SA (NIT) has originated). This design, as shown in Figure 1, has considerably reduced the FPN and suppressed the image lag [4,5,6]. Commercial products have been successfully developed for different markets.

The sensitivity of such pixels, even highly improved compared to that of traditional logarithmic pixels, is only equivalent to 3-transistor (3T) CMOS active pixel sensor (APS), which is not enough for applications that demand low light performance such as video surveillance. The fundamental obstacle to a higher sensitivity is the KTC noise caused by the reset transistor exactly as in a 3T pixel. In today’s 4-transistor (4T) pixels, this KTC noise has been suppressed by using a fully depleted buried photodiode and charge transfer readout associated with a correlated double sampling mechanism. We think that we should follow the same path to make a low-noise logarithmic pixel.

Therefore, we have investigated a new pixel design called QLog where the logarithmic compression is applied directly to the collected charge, similar to what happens inside solar-cell mode photodiodes. The fundamental difference is that the photodiode here is fully depleted at the beginning of the exposure and the residual charge in the photodiode after exposure is measured. This complete depletion possibility of the buried photodiode, associated with the charge transfer mechanism, permits the suppression of KTC in low light conditions. In high light conditions, the logarithmic compression at the charge collection period considerably reduces the total electron number required to cover a wide dynamic range. Compared to the initial solar-cell mode photodiode, the buried photodiode is not reset at the equilibrium state, but at the free-carrier empty state. By consequence, an initial linear response will be observed before the carrier-in and carrier-out reaches the equilibrium. The QLog pixel will exhibit a composed linear–logarithmic photo-electric response. The paper published at IISW2017 [7] indicates that this QLog pixel can be an elegant solution to overcome the too-low integration well capacity in actual high-sensitivity, subelectron readout noise sensors [8,9,10,11,12].

The QLog pixel has the same conceptual structure as a conventional 4T pixel with buried photodiode and charge-transfer-based readout. The transfer gate will be biased in two states: on and off. The residual subthreshold current under the transfer gate (TX) should be suppressed. This is very different to the well-known implementation of Lin–Log conversion in 4T pixels by using the TX gate in subthreshold mode [13]. In such an implementation, the silicon surface under the TX gate is not fully accumulated and a large dark current and nonuniformity will be generated, which considerably reduce the low light performance and image quality. In QLog implementation, the TX gate is fully accumulated during exposure and the logarithmic charge compression is done only by electron evaporation (thermionic emission) in the bulk. Therefore, the low dark current of pinned photodiode (PPD) can be conserved and better uniformity can also be expected. The evaporated electrons will be collected by an anti-blooming drain in order to reduce the pixel-to-pixel crosstalk.

In the following sections of this paper, we will focus on the basic logarithmic charge compression and introduce a theoretical model based on the electron evaporation phenomenon from a potential well. The influences of temperature and exposure time on the response curve of the QLog pixel will be studied by numerically resolving this theoretical model. Then we will present measured results from a test chip with 320 × 240 QLog pixels in order to validate the theoretical model and check the response uniformity across the pixels in an array.

## 2. QLog Pixel Photo-Electric Response Modeling

**Figure 2:**
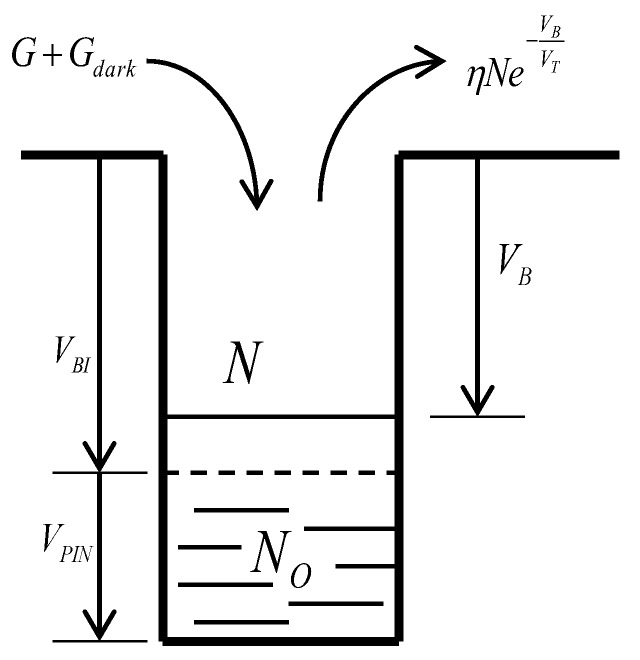
Free electron movements inside a buried photodiode.

As shown in Figure 2, a buried photodiode can be modeled as a potential well with $N_{0}$ free electrons at zero bias. In order to deplete these $N_{0}$ electrons, we should reversely bias the photodiode at voltage $V_{PIN}$ (called pinning voltage). The potential barrier height, from the bottom to the top of this potential well, is the sum of the pinning voltage and the junction build-in voltage, which is $V_{PIN}+V_{BI}$.

Due to the slow logarithmic evolution of the voltage across the photodiode junction and the very low pinning voltage used in our design, we can suppose with reasonable precision that the buried photodiode has a constant junction capacitance of $C_{PD}$. Then we can have

(1) $$V_{PIN}=\frac{qN_{0}}{C_{PD}}.$$

It is known that an electron inside this potential well has a probability to escape; this probability can be written as

(2) $$P_{r}=\eta e^{-\frac{V_{B}}{V_{T}}},\;\mathrm{where}\;V_{T}=\frac{kT}{q}.$$

The structure constant η can be fixed by using the equilibrium of the buried photodiode in darkness where the dark-generated electron number must be equal to the escaped electron number:

(3) $$N_{0}\eta e^{-\frac{V_{B}}{V_{T}}}=G_{dark}\Rightarrow \eta =\frac{G_{dark}}{N_{0}}e^{\frac{V_{BI}}{V_{T}}}.$$

Suppose that incident light generates *G* electrons per second; the total electron number inside the potential well will be governed by the following differential equation:

(4) $$\begin{array}{l} dN=(-N\eta e^{-\frac{V_{B}}{V_{T}}}+G+G_{dark})dt,\\ V_{B}=V_{BI}+V_{PIN}-\frac{qN}{C_{PD}}. \end{array}$$

**Figure 3:**
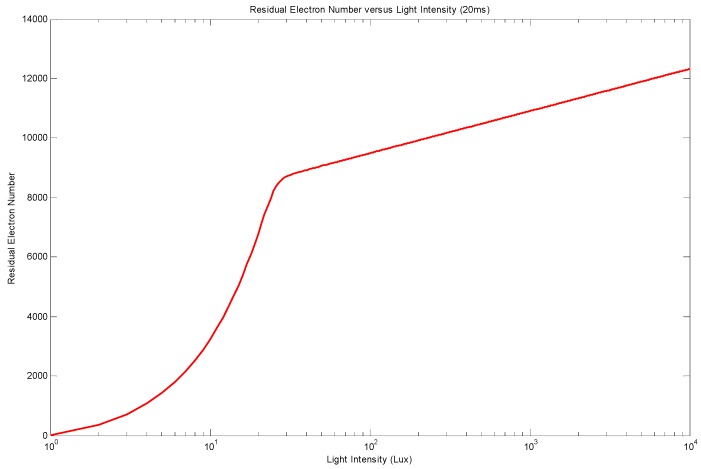
Numeric solution of Equation (4) with $V_{BI}=0.8\;V,\;V_{PIN}=0.1\;V,\;C_{PD}=4\;\mathrm{fF},\;G_{dark}=10\;e/s,$ and $T_{EXP}=20\;\mathrm{ms}$. The transition point calculated from Equation (5) is at 8300 electrons, which fits well with this numeric solution.

It is not possible to have a closed-form solution of Equation (4), but a numeric resolution can be applied. By using realistic parameters from a common CMOS process, such as $V_{BI}=0.8\;V,\;V_{PIN}=0.1\;V,\;C_{PD}=4\;\mathrm{fF}$ and $G_{dark}=10\;e/s$, the residual electron number versus light intensity is shown in Figure 3. This residual charge number with a fixed integration time follows two regimes: (1) linear regime at low photo flux and (2) logarithmic regime at high photo flux. A sharp transition connects these two regimes together.

We can calculate the linear–logarithmic transition point by using the cross point between a pure linear response with a fixed exposure time and a pure logarithmic response with infinite exposure time:

(5) $$\begin{array}{l} N\eta e^{-\frac{V_{B}}{V_{T}}}=\frac{NG_{dark}}{N_{0}}e^{-\frac{V_{PIN}}{V_{T}}+\frac{Nq}{C_{PD}V_{T}}}=G+G_{dark}\approx G,\\ N=(G+G_{dark})T_{EXP}\approx GT_{EXP}. \end{array}$$

By resolving (5), we have

(6) $$N_{LIN2LOG}=[V_{PIN}-V_{T}\ln\frac{T_{EXP}G_{dark}}{V_{PIN}C_{PD}}]\frac{C_{PD}}{q}.$$

**Figure 4:**
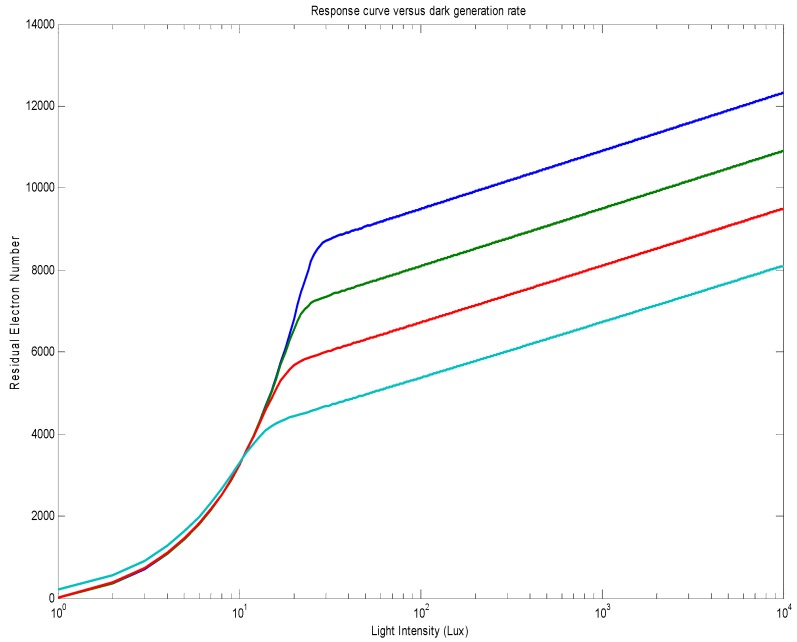
Response curves for different dark generation rates (10 e/s, 100 e/s, 1000 e/s, and 10,000 e/s in order from top to bottom). The same buried photodiode parameters as in Figure 3 have been used. The linear–logarithmic transition points match well the values calculated from Equation (6).

The impact of the dark generation rate (dark current) on the response curve is shown in Figure 4. A higher dark generation rate will generate a proportional positive offset in the linear regime and logarithmically negative offset in the logarithmic regime. Taking into account that the dark generation rate is an exponential function of temperature, the temperature change will generate a temperature–linear negative offset on the logarithmic response, the same as in solar-cell mode pixels. The logarithmic regime will arrive earlier with a higher dark generation rate.

**Figure 5:**
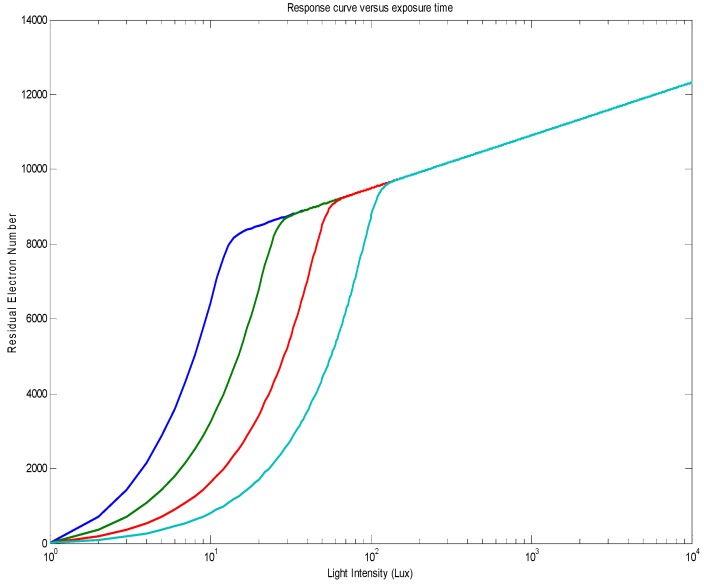
The response curve as a function of different exposure times. Here the buried photodiode parameters are the same as those in Figure 2; the exposure times are set at 40 ms, 20 ms, 10 ms, and 5 ms, respectively (in order from left to right).

The exposure time will not change the logarithmic response, but will impact the linear response so the linear-to-logarithmic response transition point is as predicted by (6). The numeric simulation result is shown in Figure 5. Since the QLog pixel has a huge native dynamic range, the exposure time can normally be fixed during the usage. However, the dark-generation-induced offset should be taken into consideration and a numeric compensation is needed in order to get a temperature-independent photo-electric response.

Generally speaking, this linear–logarithmic response exists in all potential-well-based photo-detectors. However, in a traditional pixel design, the barrier height of the collecting potential well is set as high as possible in order to minimize the nonlinearity caused by electron evaporation, so the logarithmic regime is invisible since it exceeds the designed signal excursion. The traditional dynamic range extension is made either by increasing the potential well volume, by some adaptive potential well volume adjustment, or by controlling the integration time. In our approach, the barrier height of the potential well is lowered so much that the electron evaporation phenomenon can generate a natural logarithmic charge compression with a huge useful dynamic range.

Our original solar-cell mode pixel design can be seen as a QLog pixel where the buried photodiode is prefilled by the zero initial bias voltage. The prefilled free electrons considerably accelerate the electron evaporation process and a pronounced logarithmic response is observed even in low light conditions. However, in the case of QLog, the potential well is fully depleted at the beginning of exposure and the electron evaporation is increased progressively when the potential well is filled with photo-generated electrons, giving a linear response in low light conditions. The reduced free electron number in QLog pixels has also a consequence on the barrier variation slope as a function of light intensity. In the case of QLog, it is around 57 mV/decade, slightly smaller than 60 mV/decade. The study in [14] gives more details on this phenomenon.

## 3. Noise Modelling, Dynamic Range, and Single Electron Detection Possibility

The noise modelling of this pixel design can be made separately for the linear and logarithmic regimes. When the pixel is in the initial linear regime, the noise is simply composed of collected carrier shot noise and readout noise. By ignoring the readout noise here for simplicity, this noise in electrons can be written as

(7) $$n_{e}=\sqrt{N}.$$

When the pixel is in the logarithmic regime, the carrier movement will be symmetric and the noise becomes Johnson noise. In this case, the noise in electrons can be written as

(8) $$n_{e}=\frac{\sqrt{kTC_{PD}}}{q}.$$

In the linear regime, the signal to noise ratio can be simply the ratio between the signal electron number and the shot noise electron number (again for simplicity we ignore the readout noise here). The peak SNR will be reached when the potential well of the buried photodiode is filled to the Lin–Log transition point according to (5):

(9) $$SNR_{Lin\_\max}=\sqrt{[V_{PIN}-V_{T}\ln\frac{T_{EXP}G_{dark}}{V_{PIN}C_{PD}}]\frac{C_{PD}}{q}}.$$

However, in the logarithmic regime, we shall adopt the contrast-based signal to noise ratio which is the ratio between the incremental signal electron number generated by a predefined contrast—one decade, for example—and the noise electron number ($C_{PD}$ in fF):

(10) $$SNR_{LOG10}=V_{T}\ln10\sqrt{\frac{C_{PD}}{V_{T}q}}=29\sqrt{C_{PD}}.$$

The total electron number inside the buried photodiode to cover a 120 dB dynamic range can be calculated by using

(11) $$N_{TOTAL}=N_{LIN2LOG}+\frac{C_{PD}V_{T}}{q}\ln(\frac{1,000,000}{N_{LIN2LOG}}).$$

For a buried photodiode with $V_{PIN}=0.1\;V,\;V_{BI}=0.8\;V,\;C_{PD}=1\;\mathrm{fF},\;T_{EXP}=20\;\mathrm{ms},\;G_{dark}=100$, the peak linear $SNR_{Lin\_\max}$ will be 39 (32 dB) and $SNR_{LOG10}$ will be 29 (29 dB), and the total electron number to cover 120 dB will be about 2600. If we suppose that the voltage excursion on the floating diffusion node is limited to 1 V, then a conversion gain of 384 µV/e can be used. In order to get a readout noise of 0.28 e, the source follower (SF) noise should be less than 107 µV. This is possible with a high-performance CMOS image sensor (CIS) process and clever circuit designs [15]. This result shows that we can obtain subelectron readout noise and keep a huge 120 dB dynamic range at the same time. The overall SNR is lower than the peak SNR of a traditional linear-mode CIS. It should be understood that for QLog pixels the SNR is constant over the whole dynamic range but the peak SNR in a linear pixel is only available just before the saturation of the pixel. The average SNR obtainable from a well-controlled and exposed linear image sensor is about 30% of the peak SNR. It should be noted also that the output image from QLog can be used directly in any vision applications and is adequate for most visualization purposes. Except for in some very extreme cases, local tone mapping is not necessary.

From Equations (9) and (10), it is clear that the incremental SNR in the logarithmic regime depends only on the buried photodiode capacitance and the SNR in the linear regime is determined by the Lin–Log transition point. The optimization of the buried photodiode should be directed to have a low $V_{PIN}$ and a large $C_{PD}$.

## 4. Prototype Test Chip and Measurement Results

We developed a buried photodiode and an efficient transfer gate process option in a standard 0.18 µm CMOS process. This test chip was run many times over 3 years in order to define the correct recipe, including implant dose/energy and the associated thermal process. The final process gives a pinning voltage around 150 mV and photodiode capacitance of 0.4 fF/µm^2^. A test chip with 320 × 240 pixels was designed and fabricated. The pixel pitch is 11.2 µm without optimization. The buried photodiode area is 13 µm^2^ and the floating diffusion node capacitance was designed at 4 fF. The conversion gain was set voluntarily low in order to avoid any possible saturation in the readout chain. The total readout chain has a unity gain except for the in-pixel source follower which has a gain about 0.8. The output analog signal was digitalized by using a 14-bit ADC on the test board and the AD conversion range was set at 2 V. The main purpose of this test chip was to validate our theoretical model and it was not targeted for extreme low noise performance.

**Figure 6:**
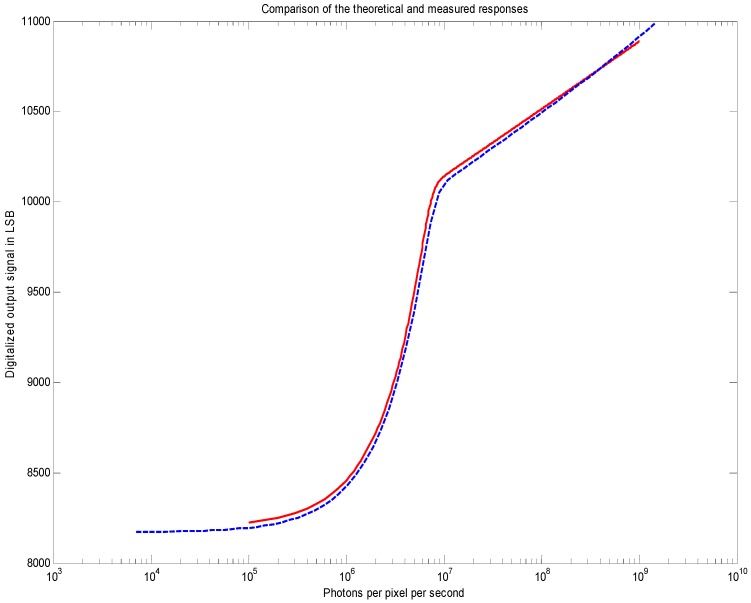
The measured response curve (blue dashed) superposed with the calculated response from Equation (4). The exposure time was set at 20 ms and estimated quantum efficiency was about 5% (Fill-Factor = 8%). The operating temperature of the sensor was 25 °C.

**Figure 7:**
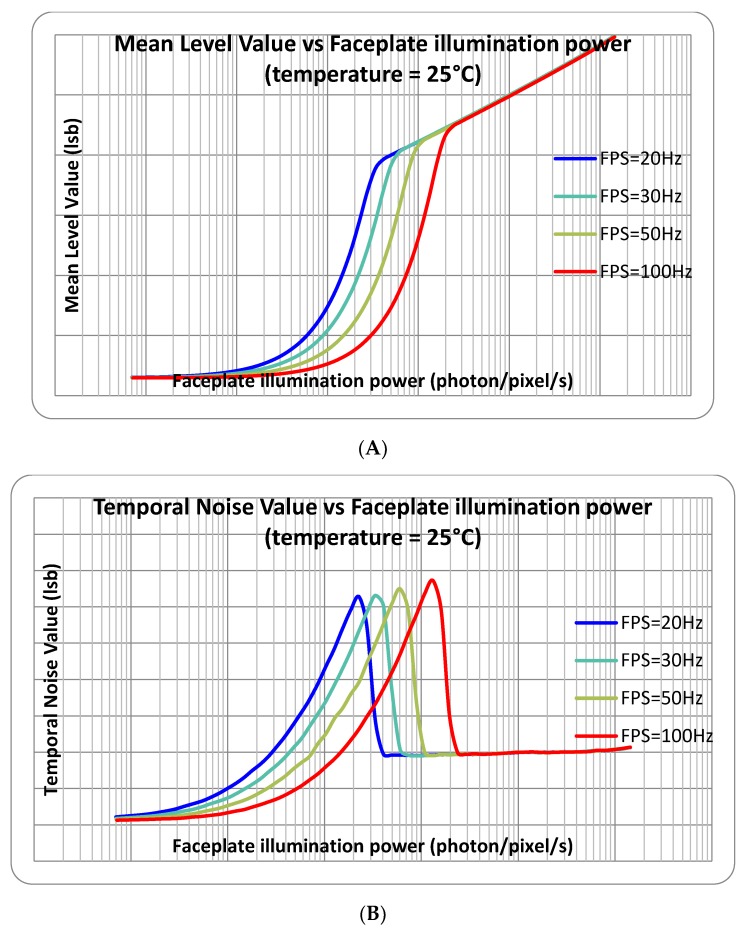
Measured photo-electric responses (**A**) and temporal noises (**B**) from the test chip at room temperature with different exposure times.

**Figure 8:**
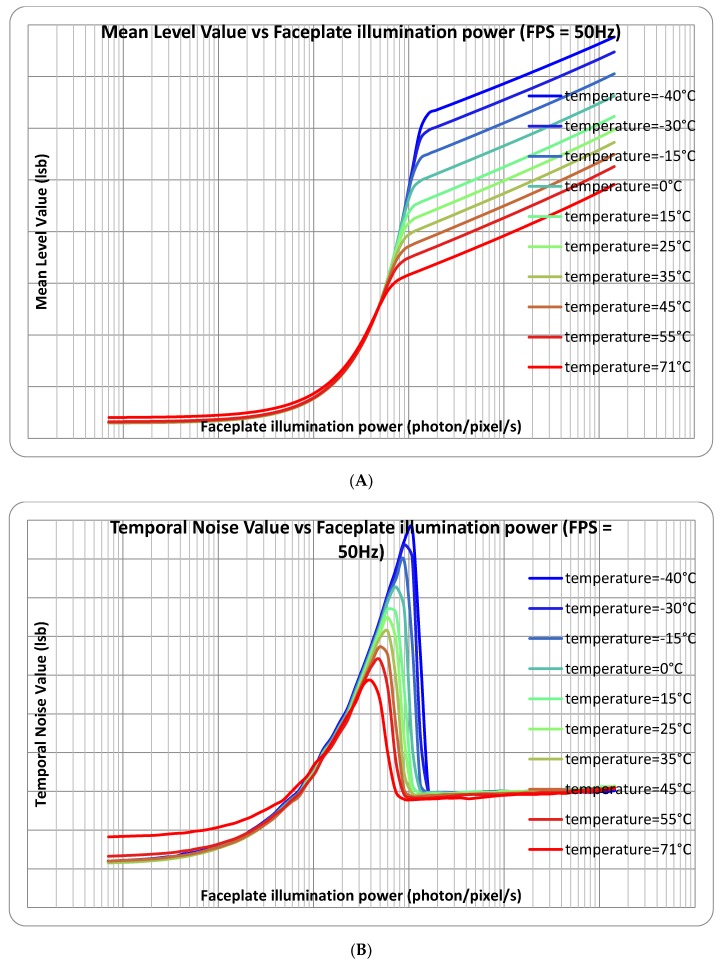
Measured responses (**A**) and temporal noises (**B**) from the test chip at 50 Hz with different temperatures.

Figure 6 shows the measured response curves at 20 ms exposure time and simulated response with the estimated pixel parameters. The linear-to-logarithmic transition matches the theoretical prediction. The pinning voltage (150 mV) was measured by using a JEFT transistor made with buried photodiode implants. The buried photodiode capacitance (5.2 fF) was measured by using Johnson noise in the logarithmic regime. The matching between these responses is very good. Figure 7 and Figure 8 show the measured response and temporal noise with different exposure times and different temperatures.

The readout noise was measured at 2.2 LSB, which is 268 µV. Taking into account the source follower gain, the temporal noise on the floating diffusion was estimated at 335 µV. With a floating diffusion node capacitance estimated from design at 4 fF, the noise electron number is 12.3 electrons. The temporal noise in the logarithmic regime was measured at 6 LSB, which represents 34 electrons inside the buried photodiode. From this Johnson noise, the photodiode capacitance can be estimated at 6.2 fF which is quite close to the estimation from the layout.

**Figure 9:**
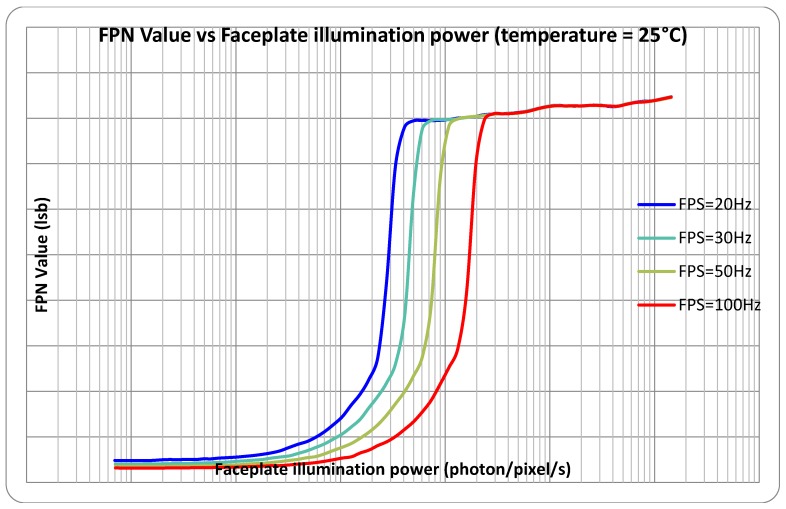
Measured fixed-pattern noise from the QLog test chip at different exposure times.

The fixed-pattern noise was measured and is shown in Figure 9. We can see that there is a significant FPN when the QLog pixels go into the logarithmic region. It represents 10% of the signal with a decade of contrast and without correction, the image quality is poor. It can be seen that this logarithmic fixed-pattern noise is nearly constant over the whole logarithmic range, which indicates that the main source should be the barrier height variation. We are still investigating the sources of this high FPN, from both the circuit design and the CMOS process recipe.

## 5. Conclusions

We have presented the new logarithmic pixel design QLog with wide dynamic range and low noise. This design can be seen as a charge domain extension from NIT’s solar-cell mode photodiode logarithmic pixel design. This new design benefits from a fully depleted buried photodiode and charge-transfer-based readout in order to remove the KTC noise at low photon flux. The logarithmic compression at the photo-generated carrier collection stage limits the number of carriers necessary to cover a wide dynamic range. This highly reduced carrier number gives the possibility to use ultra-small capacitance for the floating diffusion to get a high enough conversion gain and overcome the noise of the source follower transistor in order to obtain single carrier detection capability. The theoretical modelling has been validated in silicon. The test chip has shown both low noise and an ultra-wide dynamic range. The residual FPN in the logarithmic regime, the exposure–time, and the temperature-dependent Lin–Log transition are currently under investigation.
